# Dental Alloys

**Published:** 1877-08

**Authors:** Henry S. Chase


### ARTICLE IX.

### *Dental Alloys.*

BY HENRY S. CHASE, M. D.

Written for the Missouri State Dental Society.

During the last year I have experimented in the making of dental alloys. I have desired an alloy that would not darken to a disagreeable extent. Some excellent alloys discolor considerably in the presence of acid saliva. The substances of which plugs are made should be, in a measure as subject to chemical disintegration as the dentos in which it is placed. If an alloy plug darkens on its surface it shows that it has been subject to a chemical cause. To be sure, what may darken the surface of a plug might not injure dentos. For instance, sulphuretted hydrogen might darken a plug owing to a sulphide of silver being formed. The sulphuretted hydrogen would not disintegrate dentos. But some acid conditions of the mouth will darken the ordinary alloys Almost any of the alloys, that do not contract, are good preservers, if properly manipulated. Alloys *not* containing gold are liable to contract, or change their forms in plugs *weeks* after they have been used, although they may not in 72 hours. Gold controls contraction and expansion also. It controls also, in a measure, discoloration.

. As *silver* is at present indispensable to an alloy plug, more or less discoloration must be expected in some mouths. But, in order to obtain the best results in the present condition of our knowledge, I have instituted hundreds of experiments. I assure you these experiments have required much time and patience, and have resulted in frequent disappointments, during the time occupied. The melting of metals is a simple enough thing; but their union in such proportions as is most desirable is neither simple, or *easy*, even after one "knows how." My experiments have at last resulted in three alloys which have been tested both in and out of the mouth many hundreds of times since last October.

I will designate these as Nos. one, two, and three:

FORMULA No. 1.—Gold, $33\frac{1}{3}$; Silver, $33\frac{1}{3}$; Tin, $33\frac{1}{3}$ This is a very quick-setting alloy, becoming exceedingly hard in a few minutes. It requires from 60 to 80 per cent. of quick silver. Chemically pure tin *filings* may be added to this, if desired, in certain proportions.

To 100 parts by weight of "No. 1," may be added 25 parts of tin filings. The effect is to take a less per centage of quick silver; to delay hardening, and to render the plug *less* tough. A less quantity of tin filing may be used, but not greater. A greater will make a plug defective by the "tube test."

FORMULA No. 2.—Gold, 25; Silver, 39; Tin, 36. This is less quick in setting than "No. 1," makes a sufficiently hard plug, and requires from 40 to 60 per cent. of quick silver. No *tin filing* can be added to this without injury.

FORMULA No. 3.—Gold, 20; Silver, 40; Tin, 40. This is more plastic and requires more time to harden than "No. 2." It requires from 30 to 50 per cent. of quick silver. It will not bear the addition of *tin filings.*

All of these alloys keep their color remarkably well—even under unfavorable conditions. All stand the "tube test" for not only days, but for months.

None but *chemically pure* metals must be used. None must be burned out in melting—at any rate, not more than half a grain in 100 should be lost in melting, or one-half of one per cent.

To do this requires skill, care, and experience. You can get the experience if you try as many times as I did before I succeeded. I procure a nice piece of charcoal that will not *snap*. Cut a basin in the end and use this for both crucible and ingot. Melt the gold first, then add the silver, when the latter is fluid and mixed with the former, add the tin. Stir well with a clean and *polished* steel wire. Cool as suddenly as possible by holding a cold steel hammer head on the melted surface of the alloy. After filing the alloy into powder, the particles of iron which it contained must be removed by a magnet.

These alloys may be washed while mixing with quick-silver. *Nothing* but *alcohol* is allowed. They should be mixed in the palm of hands, and *not* in a mortar, as *less* quick-silver is requited by the former method.

If washed, *perfect dryness* must be attained before using. This can be done by washing before adding *all* the quick-silver, and by making fine grains of the mass while blowing upon it. After it is dry, the balance of the quick-silver may be added.

These alloys are not to be used excepting in *dry* cavities.

I have freely given you now the results of many months' labor. If you are as successful as I have been in making and using these alloys, you will not regret it. If you fail, you may wish you had never made the trial.—*Missouri Dental Journal.*

---

